# A comparative analysis of the impact of online, blended, and face-to-face learning on medical students’ clinical competency in the affective, cognitive, and psychomotor domains

**DOI:** 10.1186/s12909-022-03777-x

**Published:** 2022-11-01

**Authors:** L. C. Enoch, R. M. Abraham, V. S. Singaram

**Affiliations:** School of Clinical Medicine, Clinical and Professional Practice, College of Health Sciences, University of KwaZulu-Natal, Durban, South Africa

**Keywords:** Online learning, Knowledge transfer, Theory-practice gap, Clinical skills, OSCE, COVID-19 pandemic, Medical Education, Blended learning

## Abstract

**Background:**

The Coronavirus Disease-2019 (COVID-19) pandemic in South Africa compelled medical schools to switch to a purely online curriculum. The innovative changes transformed the standard clinical skills curriculum to increase learning transfer to bridge the theory-practice gap. The efficacy of this intervention remains unknown. This study aims to measure medical students’ clinical competency in the affective, cognitive, and psychomotor domains by assessing clinical skills knowledge retention and transfer from the online platform compared to face-to-face and blended learning.

**Methods:**

A non-random cross-sectional quasi-experimental study assessed third-year medical students’ knowledge retention and learning transfer in three domains of clinical skills competence. Data were obtained using a score sheet during a directly observed formative and a trial online summative assessment. One hundred and one third-year medical students volunteered for the formative onsite assessment that tested the psychomotor domain. Two hundred and thirty-nine students were evaluated on the affective and cognitive domains in the summative online trial mini-objective structured clinical examination (tm-OSCE). The OSCE scores were analysed using descriptive statistics. The significance of the findings was evaluated by comparing OSCE scores with the pre-pandemic 2019 third-year medical students.

**Results:**

Statistically significant differences were found between the two cohorts of medical students from both years (p < 0.05). The 2021 blended group’s (n = 101) medians were 90%, 95%CI [86, 92], 82%, 95%CI [80, 85], and 87%, 95% CI [84, 90] for the psychomotor, affective, and cognitive skills, respectively. The e-learning group’s affective and cognitive skills medians were 78%, 95%CI [73, 79] and 76%, 95%CI [71, 78], respectively. The 2019 face-to-face cohort (n = 249) achieved medians of 70%, 95% CI [69, 72] and 84%, 95%CI [82, 86] for the affective and psychomotor skills, respectively.

**Conclusion:**

Medical students demonstrated near and far transfer bridging the theory-practice gap in three clinical skills domains. The blended group performed significantly better than the e-learning and face-to-face groups. Medical schools and educators play a vital role in overcoming learning challenges and achieving higher transfer levels by adopting multiple student-centered teaching delivery approaches and arranging immediate application opportunities. This study offers medical educators suggestions that encourage the transfer of online learning to face-to-face practice, decentralising medical education with a revised blended learning strategy.

## Introduction

In March 2020, medical education transitioned drastically in South Africa due to the SARS-CoV-2 pandemic. With minimal preparation, educators and students had to adapt to the unconventional practice of teaching and learning “hands-on” clinical skills on a “hands-off” online platform [1, 2]. Conventional teaching methods are structured as face-to-face interactions in a clinical skills laboratory [3]. However, this was no longer possible due to the physical distancing measures stipulated during the pandemic. This sudden change demanded innovative teaching strategies and entirely transitioned to online learning [4] to ensure the continuity of undergraduate clinical skills training [5–7].

### The traditional teaching of clinical skills (pre-pandemic period)

Clinical skills are traditionally taught in a clinical skills laboratory (CSL) using mannequins or simulated patients trained to pose as examination models. Training supports the combination of practical examination, procedural and communication skills, and patient management [8] to develop competence in the psychomotor, affective, and cognitive domains of clinical skills [9], respectively. Bloom’s classification considered that demonstrative, behavioral, and intellectual knowledge could be stratified within the three domains [10]. Teaching these skills has evolved from Halsted’s apprenticeship model [11] of “see one, do one” to a constructivist model where students actively build their knowledge on an existing foundation [12]. Online learning has been increasingly integrated into medical education through learning management systems (LMS), where students can pre-read content before synchronous lectures are delivered. This encourages self-paced learning of core knowledge to aid robust engagement in the classroom [13]. The practical application is taught in the CSL – a safe space for students to learn the fundamental practices in a standardised and controlled environment compared to a hospital setting, where actual patients may impact the student’s ability to learn a skill for the first time [14]. However, the traditional learning environment does not entirely satisfy students’ needs. Skills have varying levels of difficulty, time is restricted, and student cohorts are large, impacting the effective transfer of learning and knowledge retention needed for students to become competent [15, 16].

### Transfer of learning

Education aims to apply what we learn to different contexts and extend this learning to new situations, thereby bridging the theory-practice gap through transfer [17, 18]. Transfer of learning occurs when existing knowledge, skills, and abilities affect the learning and performance of new tasks [19]. A notable difference between “learning” and “transfer” is that “learning” implies that the same task is repeated, whereas “transfer” implies that the task may differ in varying degrees from the original context [20].

Thorndike’s “Identical Elements” theory states that two tasks may differ yet share common components [20]. Therefore, transfer theories can be described as near or far. Near transfer is when the context of the assessed task resembles the initial learning situation. Far transfer is when the original learning context differs from the application environment [17]. The online teaching and learning environment is vastly different from the eventual application context at the bedside[21]. Therefore, to optimise the transfer and retention of knowledge and skills from the online platform, the salient details of the skill must be identified and taught [19].

### Retention

Knowledge retention is essential for diagnostic decision-making in medical practice. Doctors execute this skill primarily due to clinical reasoning, as described by the Dual Process theory. The theory comprises System 1, which is fast, autonomous, and expertise-driven, and System 2, which is slow, analytical, and uses higher-order processes [22]. Students employ the latter due to their lack of experience in medical practice. In place of experience, clinical reasoning and enhanced decision-making rely on knowledge recollection. Retention, however, is a struggle for many students, as shown by the Ebbinghaus Forgetting Curve [23]. A typical “forgetting curve” suggests that 50% of new knowledge is forgotten by learners just twenty minutes after the lesson has ended [24]. However, as learners absorb new and profound concepts more meaningfully, it is expected to be forgotten more slowly [25], augmenting a cognitivist model. “Hands-on” training in a CSL promotes deliberate practice, further developing expertise and competence [26]. The complete transition to the online platform may deprive students of the experiential learning opportunities associated with the face-to-face curriculum [27, 28].

### The transition to online learning (pandemic period)

In the United States of America, the Instructional Technology Council has defined E-learning as delivering instructional materials to remote sites using technologies such as the internet and smart devices [29]. The online transition of medical education saw institutions of higher learning using videoconferencing platforms like Zoom and Microsoft Teams to deliver lectures [28, 30–32]. Besides the challenge of losing in-contact training, novel challenges also arose. Globally, students and tutors faced early issues in transitioning to this platform due to technical naivety, poor connectivity, and device issues [33, 34]. In developing countries, the situation was compounded by other socio-economic challenges, such as the lack of infrastructure and financial support [35]. Additionally, South Africa experienced an unstable electrical grid resulting in rolling electricity outages called “load shedding,” causing further difficulties.

Although the global pandemic catapulted medical education into the online realm, the medical educator’s goal remains to develop learners into critical thinkers capable of clinical reasoning skills which is the hallmark of the competent physician [22]. There are concerns about the retention and transfer of learning from online training settings to the clinical skills laboratory in this unprecedented situation, making it necessary to identify students’ knowledge gaps to correct incompetence. While tutors’ and students’ perceptions of online and blended learning have been vastly researched [36, 37], the effectiveness of this intervention as a sole pedagogy in medical education has not been well established [1, 38]. Brabrand [39(p1)] asked, “How can we make sure our students learn what we want them to?” Similarly, the authors of this study ask: “How can we be sure that the students learned the clinical skills we taught them in the new online teaching platforms?” This understanding will inform post-pandemic pedagogical changes and practices regarding online versus face-to-face or a combination thereof. Hence this study aims to evaluate medical students’ capacity to bridge the theory-practice gap by assessing their retention and transfer of clinical knowledge and skills following the adapted online and blended training programme in comparison to a previous face-to-face programme.

## Methods

### Setting and Context

This study was conducted at the Nelson R Mandela School of Medicine, University of Kwa-Zulu Natal, during the second semester between August and November 2021. Medical students at this institution complete their pre-clinical training in their third year in the CSL using simulated patients before beginning clinical practice at the hospital with actual patients. During the pandemic, all teaching at the University of KwaZulu-Natal medical school was conducted off-site for more than one year using the online Zoom videoconferencing platform. Tutors delivered live interactive clinical skills lectures and “practical” sessions synchronously, while pre-recorded lectures were uploaded onto the Learning Management System – Learn2021® asynchronously. Teaching was adapted for the online platform, and all lessons were conducted remotely. Students had access to tutors via email, discussion forums, and interactive Zoom sessions.

#### Adaptations to the clinical skills online teaching and assessments

- Psychomotor/Examination and Procedural Skills:

The online Zoom training for examination and procedural skills was conducted by adapting and modifying George’s [40] five-step framework for teaching clinical skills (Table 1)[6].

Step 1 is the overview, addressing the need for the skill. Traditionally this was taught in a lecture hall. For online learning adaptation, voice-recorded lectures were uploaded onto Learn2021® as pre-reading material or delivered synchronously as an online live lecture over Zoom. Each method offered advantages to learning. Asynchronous pre-recorded lectures allowed students to pause, rewind, or revisit the lecture repeatedly, while synchronous, interactive lectures promoted tutor engagement and immediate clarification as needed.

In Step 2, the preceptor demonstrates the skill precisely without explanation. Traditionally this was done by playing a video of the skill being performed. There was no need to adapt this step as the same videos were shared with the students for online self-directed learning.

In Step 3, the preceptor demonstrates the skill again but takes time to explain each process. In the traditional setting, the tutor demonstrates the skill on a simulated patient with a detailed explanation. The adapted step 3 replaced demonstration and explanation by the tutor with “discussion.“ This involved demonstration by video and stepwise explanation by the tutor. Additional teaching media was used to integrate the students’ existing knowledge into the new knowledge, emphasising “why” the technique was performed specifically, not merely on “how” a procedure was done.

The traditional Step 4 comprises a demonstration by the tutor and a step-by-step explanation by students. This was modified to “comprehension,“ with the demonstration by video while students explained the systematic approach and techniques.

Step 5 is where the students are allowed to practice the skill on the simulated patient while receiving feedback from their peers and tutor. This step was modified to “consolidation,” allowing for demonstration by students on themselves, where possible, or simulated models created by students at home using household items. Self-demonstration skills were restricted to body parts easily seen across the video platform. Where demonstration was not possible, students analysed pictures and videos to consolidate their learning and create a clinical context.

**Table 1:** George’s Simple Five-step Method for Teaching Clinical Skills and our adaptations in clinical skills online teaching [6]

	Traditional Method	Adapted Online Method
1	LGRS in Lecture Hall	Voice-over recording/Zoom Lectures
2	AV DEMO	AV DEMO
3	Demonstration with explanation by tutor	Discussion with stepwise explanation by tutor
4	Tutor demonstrates; students explain	Comprehension – explanation by students
5	Student demonstrates with feedback	Consolidation – linking to clinical context

b. Affective/Communication Skills:

Communication skills adaptation was achieved by virtual simulation-based training using Zoom as the online platform (Fig. 1). The Calgary-Cambridge Guide (CCG) to the medical interview [41] was screen shared and discussed. All participants had their video and audio turned on. A tutor acted as the simulated patient, a student acted as a simulated doctor, and the remaining participants observed the virtual consultation and gave feedback. The simulated doctor took a part of the history from the simulated patient as per the CCG. Throughout the case, all students provided verbal or written feedback and medical summaries on the Zoom chat facility.

**Fig. 1:**
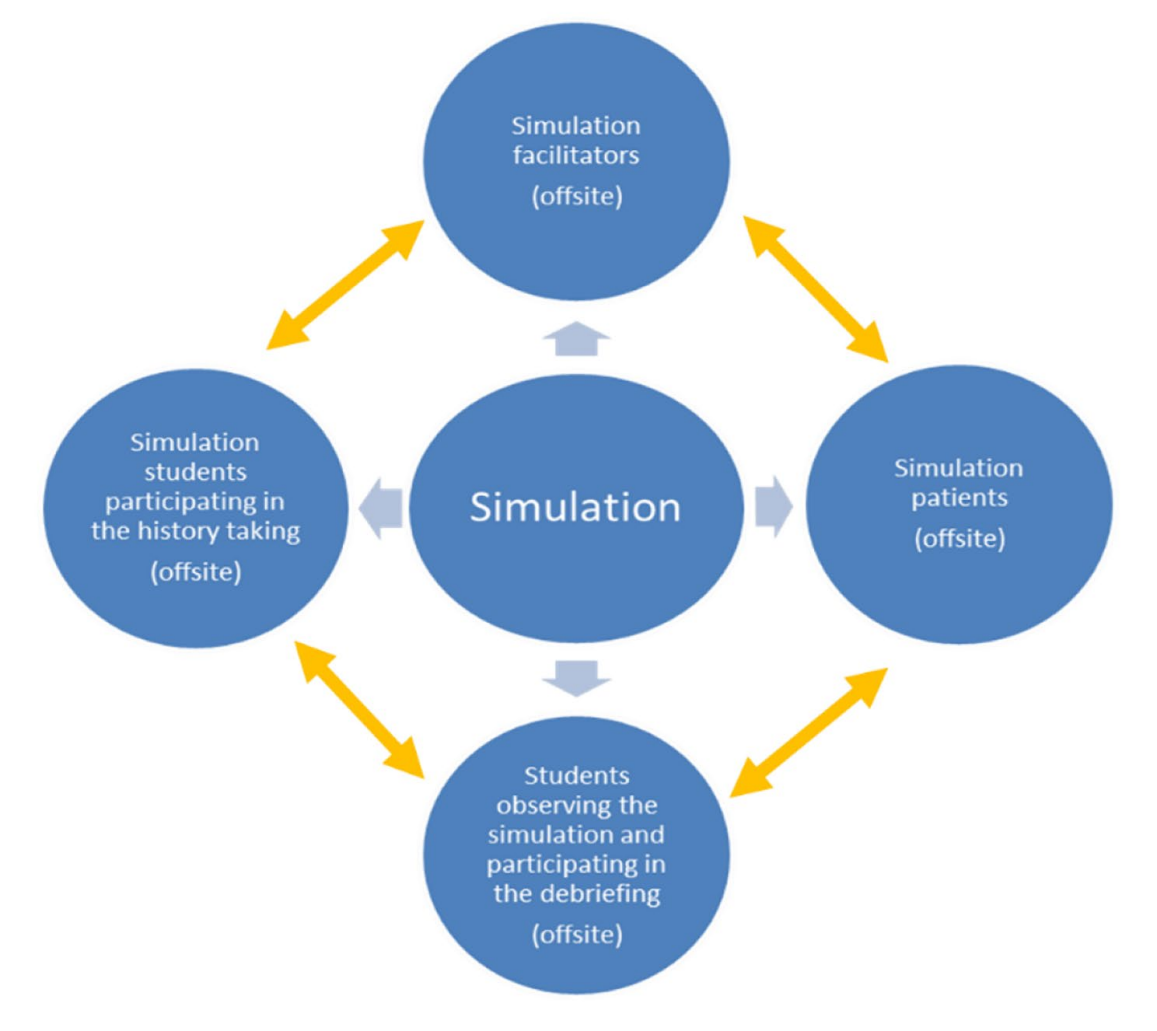
Communication skills virtual simulation process [31]

c. Cognitive Skills:

Spaced learning was implemented to aid knowledge retention by ensuring that students had multiple opportunities to interact with the same topic with repeated exposure to the material at each of the five steps (Table 1). For step one, a live zoom lecture introduced the topic, followed by videos and self-directed learning through mandatory weekly quizzes on Learn2021®, where passive feedback was given as preparation for the scheduled online practical sessions. Each topic was covered as an online practical session in smaller groups with a tutor for steps three to five. These sessions included active learning where students participated in polls, answered questions via Zoom chats, and received immediate feedback from tutors. Students then had to prepare for summative tests after each theme and an OSCE at the end of each semester. This method motivated students to revisit topics hours, days, weeks, and months after their first contact with new information, an approach that is well-recognised as effective in retaining knowledge [24, 42].

d. Standardisation and validation of assessments.

Students’ clinical competence at the NRMSM is assessed with a written test and an Objective Structured Clinical Examination (OSCE). Ten to twelve stations are assessed by experienced clinical educators. The OSCE includes stations that test professionalism, communication, physical examination, procedural, and cognitive skills. A panel of experts developed the OSCE score sheet that is task specific for each domain and is annually updated and validated. The COVID-19 pandemic posed a global challenge for clinical skill lab assessments. Many institutions opted to postpone or cancel clinical and written examinations, while others chose to introduce online methods of assessments [43]. Since the third-year end-of-semester assessment was a low-stake, non-exit module examination or a pre-requisite to pass the module for graduation purposes, the NRMSM opted to adapt the assessment strategies. The first assessment was conducted as a formative onsite examination that assessed the performance of two psychomotor skills. Due to COVID-19 risks and restrictions, there was voluntary enrolment and mandatory screening of eligible students for participation. Using the existing validated OSCE mark sheet, the assessment was conducted under examination conditions by six experienced examiners who were also clinicians involved in third-year undergraduate teaching. This assessment was followed by an end-of-semester online summative trial mini-OSCE (tm-OSCE) that was piloted to assess affective and cognitive clinical competence using three clinical skills. To standardise the tm-OSCE, six trained examiners were appointed to validate the OSCE stations by the Modified Angoff Approach [44]. The examiners were involved in teaching and OSCE assessments before the pandemic and transitioned to online teaching during the pandemic. Each assessment station measured three skills: history-taking communication, physical examination, and procedural skills. The standard OSCE mark sheet was reviewed at a consensus meeting with all educators involved in the assessment. The traditional in-person OSCE mark sheet for the history-taking skill was accepted as valid for the online examination. The psychomotor skill mark sheets were adapted to ensure content validity and reliability for the tm-OSCE using case vignettes in a viva voce format. Each skill had a separate objectives-based checklist linked to domain-specific outcomes outlined in the curriculum. A constant difficulty level was maintained for all components. A dry run of the examination was conducted to train the examiners and authenticate the process. Multiple examiners assessed the same station, and ensured consistency by utilising checklists to standardise the process [44].

## Study design

### Study population, sample size, and sampling method

A non-random cross-sectional quasi-experimental study was conducted to assess medical students’ knowledge retention and both near and far transfer of clinical skills following online training. This paper focuses on the comparative component of teaching delivery methods implemented to satisfy learners’ needs in 2021 and 2019, during and before the pandemic. It includes a prospective Cohort A (2021) who engaged in either online or blended learning, and a retrospective Cohort B (2019) of medical students who had engaged in traditional face-to-face learning.

#### Cohort A:

Third-year medical students from 2021 were selected for the study as they had experienced almost two years of exclusive online learning and are referred to as Cohort A.

After completing the online teaching curriculum, Cohort A was invited to participate in a two-hour onsite formative assessment initiative called the “Readiness Programme” (RP) a week before the online summative tm-OSCE. The RP participating students were a subgroup of Cohort A and labeled Group A1 (n = 101) or the blended group. This RP session aimed to determine if students taught online could perform the skills competently before receiving feedback and correcting techniques. No additional teaching of new material was conducted.

Students who participated in the online summative tm-OSCE but did not attend the RP were labeled Group A2 (n = 138) or the e-learning group. The blended Group A1 and e-learning Group A2 together comprised the 2021 3rd year MBChB Class (n = 239), and all participated in the end-of-semester online tm-OSCE.

#### Cohort B:

The 2019 third-year class was trained and assessed in the traditional onsite clinical skills programme, which included synchronous in-person lectures, practicals, and self-directed learning resources, before the pandemic. They are the face-to-face learners known as Cohort B (n = 249).

Qualitative data on the students’ and clinical teachers’ perspectives of the usefulness of the formative and online OSCE to assist with clinical cognition and transfer of learning were thematically analysed and will be reported in more detail in a follow-up study.

### Data collection

Data for Cohort A was collected from the formative onsite assessment (RP) and the summative online assessment (tm-OSCE). Data for Cohort B was collected from the 2019 summative onsite OSCE.

Onsite Formative OSCE:

The RP was hosted as an onsite formative OSCE to evaluate the students’ competence in specified psychomotor skills following online learning. The obstetric examination and the pap smear skills were selected as they assessed far transfer, with varying degrees of difficulty. The students were advised to prepare for the session as expected for a summative examination.

#### Conduct

At the RP, the blended-group students were allowed 15 minutes of self-directed practice to familiarise themselves with the equipment and models for the obstetric exam and Pap smear skills. They were then directed to an examiner who assessed both skills using the OSCE score sheet. Each student was allowed 10 minutes to complete both skills and was scored on their professionalism, behavioral knowledge, and psychomotor performance. The examiner offered feedback and correction on the performance. Without additional marks awarded, a second attempt at the skills was provided to clarify misconceptions.

**Fig. 2:**
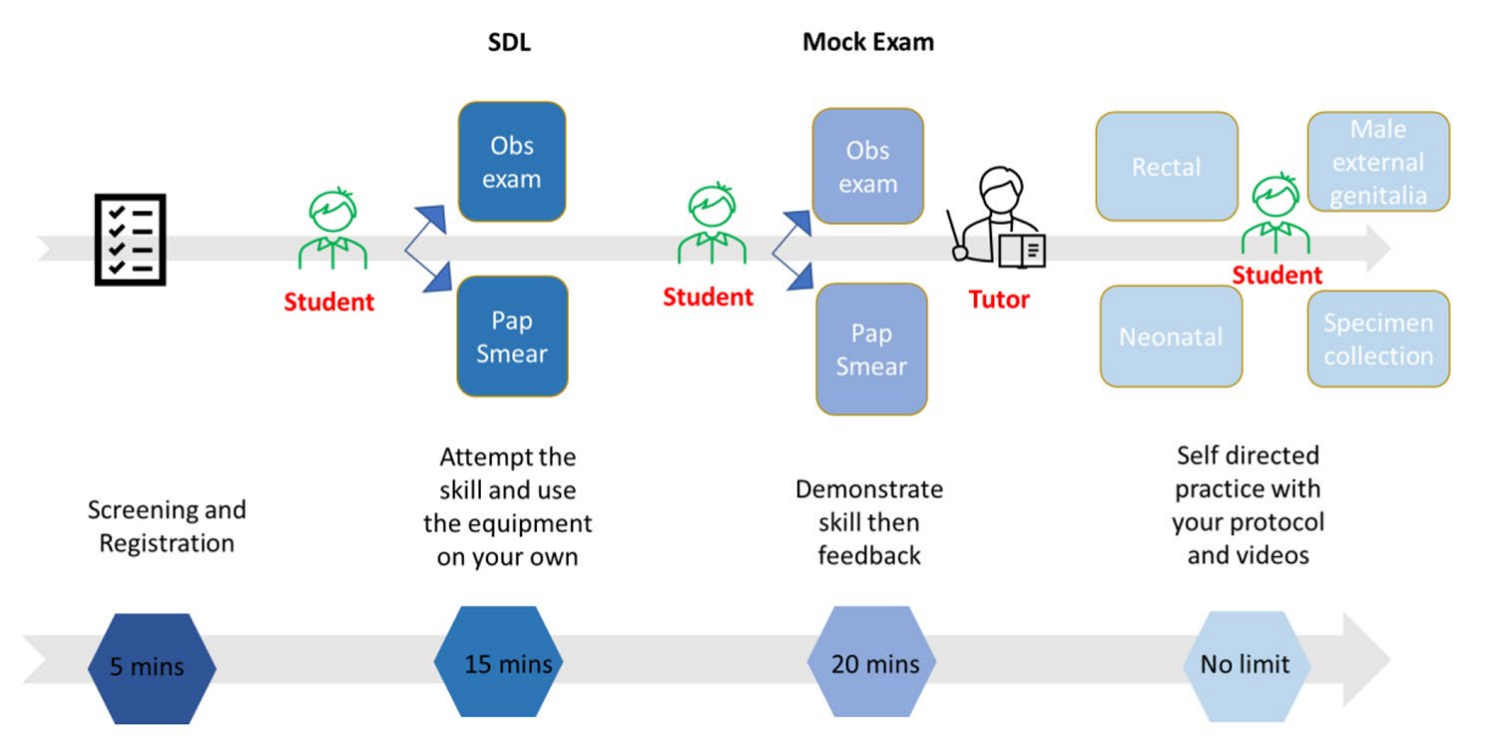
Formative OSCE process assessing psychomotor skills and data collection – Blended Group A1

Students continued with self-directed practice at other skills stations post-assessment under tutor supervision. Figure 2 illustrates the process.

#### Online Summative OSCE:

A low-stakes end-of-semester summative tm-OSCE was piloted online with Cohort A to assess affective and cognitive clinical competence using three clinical skills. All examiners and students were remote and used the Zoom videoconferencing platform. Students entered the main “meeting room” to be verified and assigned a breakout room. One examiner assessed each student on three skills in the breakout room station, minimising the risk of connectivity and technical issues associated with reconnecting to different examiners via different links.

The first skill assessed was the communication skill using a reproductive history-taking scenario based on the CCG [41]. The student had eight minutes to gather the history from the simulated patient role-played by the examiner, identify the presenting problem, background history, and the patient’s perspective of the illness using various process skills. They also proposed a differential diagnosis and answered clinical questions. This skill assessed professionalism, behavioral knowledge, clinical reasoning, and hence a combination of competence in affective and cognitive clinical skills [10]. History-taking was taught online repetitively, and the examination format closely resembled the teaching environment needed for near transfer.

**Fig. 3:**
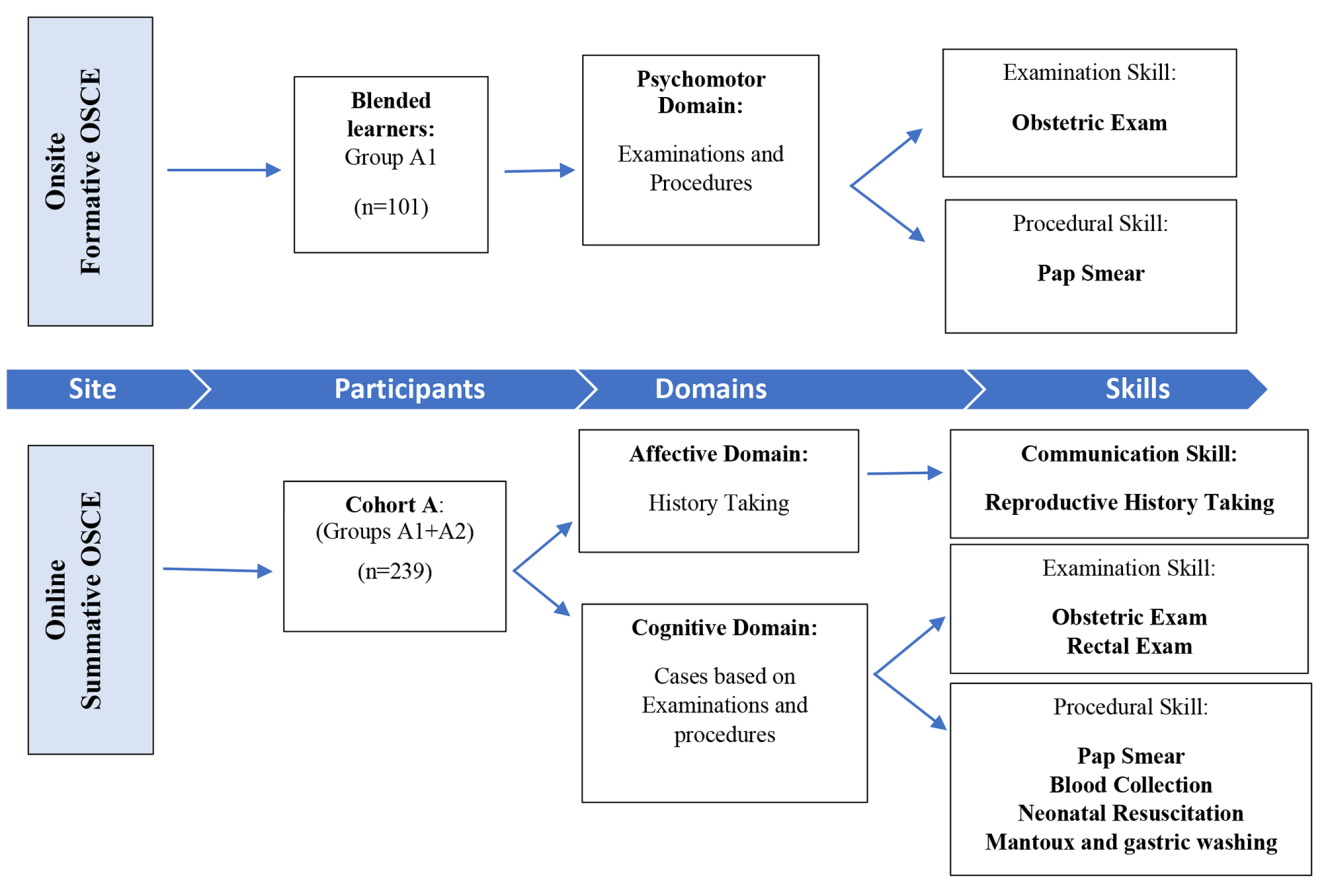
Method of assessment and data collection of 3rd -year students in 2021

The second was a five-minute physical examination skill where the student was assessed on their understanding of one of the examination scenarios, as illustrated in Fig. 3.

The third was a five-minute procedural skill assessment. The student was questioned on one of the procedural scenarios, as illustrated in Fig. 3. The physical examination and the procedural skills were assessed for near transfer using a clinical scenario where students were required to explain the indication, procedure, and principles underpinning the skill in a viva voce. These scores were combined to assess intellectual knowledge and clinical reasoning as part of the cognitive domain of skills competence [10].

As mentioned previously, standardisation of the formative and summative tm-OSCE was ensured with trained, experienced examiners appointed to validate OSCE stations by the Modified Angoff Approach [44]. Examiners evaluated students in short and long skills using simulated patients for communication skills and clinical scenarios (tm-OSCE) or mannequins (formative OSCE) for the physical exam and procedural skills. Identical stations were replicated with multiple examiners. To ensure marking consistency, students were scored on their performance of each skill by using structured mark sheets against domain-specific curriculum-based content, as in the traditional OSCE.

The data for Cohort A was collected for all three domains of clinical skills during the formative onsite (Group A1 only) and summative online OSCEs (Groups A1 and A2) held in 2021, as summarised in Fig. 3.

**Fig. 4:**
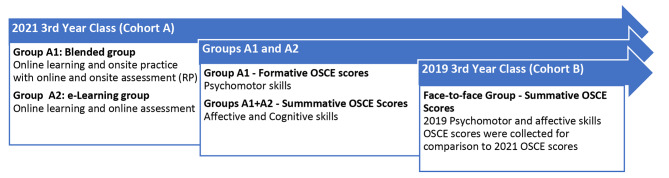
Timeline of data collection from Cohort A [blended group A1 & e-learning group A2] and Cohort B [face-to-face group]

The impact of online learning was determined by comparing the OSCE scores of online learners (Cohort A) to pre-pandemic learners (Cohort B). Cohort A’s affective/history-taking scores were compared with Cohort B’s scores, and Group A1’s psychomotor/pap smear scores were compared with Cohort B’s scores for the same skill. A summary of the data points is seen in Fig. 4 below.

### Instrument

The data collection instrument for assessing the affective and psychomotor domains of clinical skills competency was the validated pre-pandemic OSCE rubric. The scoring includes professionalism and the ability to verbally and physically demonstrate an approach to the application of knowledge while reasoning through the process. The history-taking skill was scored out of 50 marks (Annexure 1a). It assessed the extraction of the biomedical history, the process skills used for gathering this information from the simulated patient, and the ability to derive a differential diagnosis. The obstetric examination and the pap smear skill were scored out of 25 marks each (Annexures 1b and 1c) and assessed the professional approach and techniques required to perform these skills. The cognitive data collection tool was a newly developed OSCE score sheet (Annexure 1d) adapted for the online examination. It was scored out of 25 marks and assessed the application of knowledge and clinical reasoning. The validity of the instruments was pre-determined, as previously described.

### Data Analysis

The quantitative data were analysed statistically using the Statistical Package for the Social Sciences version 27 (IBM, USA). The results were interpreted using descriptive statistics to assess the medians and illustrated using graphs and tables. Confidence intervals [CIs] were set at 95% and statistical significance at *p* < 0.05. The normality of the distribution was assessed using the Shapiro-Wilk test. As the data was not normally distributed, non-parametric tests (The Mann-Whitney U test and Spearman Rho test) were used to determine the differences between the median scores and correlations between the psychomotor skills examined, respectively.

Ethical approval for this study was granted [HSSREC/00003459/2021] by the University of KwaZulu-Natal’s ethics committee.

## Results

One hundred forty-two students volunteered to participate in the formative onsite OSCE (59%). However, only one hundred one students, Group A1 (n = 101), were selected due to COVID-19 pre-screening requirements and related challenges (response rate = 42.3%). Group A2 (n = 138) made up 67.3% and included the remainder of the class. Groups A1 and A2 comprised Cohort A (n = 239) (Fig. 4). Group A1 fairly represented the diversity in the third-year class’s age, gender, and ethnicity. The demographics of the 2021 third-year class (Cohort A), the subgroup that attended the RP session (Group A1), and the 2019 third-year class (Cohort B) showed similar trends and are seen in Table 2 below.

**Table 2:** Demographics of 3rd year medical students in Cohort A (2021), Group A1 (2021), and Cohort B

DEMOGRAPHIC		Cohort A 2021 (%)	Group A1 (2021) (%)	Cohort B 2019 (%)
Age	18–25 > 25	93.9 6.1	91.1 8.9	93.4 6.6
Race	African Indian White Coloured	72.1 19.4 2.0 6.1	58.4 36.6 2.0 3.0	82.9 11.2 0.8 5.0
Sex	Male Female	46.2 53.8	43.6 56.4	49.2 50.8

### Formative Onsite OSCE

#### a) Psychomotor Domain

The median OSCE score achieved by the 2021 blended group (Group A1) for the pap smear procedural skill and the obstetric examination was 90% 95%CI [86, 92] and 84% 95%CI [80, 86], respectively. There was a correlation coefficient of 0.539 (p < 0.001) between both scores, indicating a positive correlation between the performance of both skills that required far transfer.

**Fig. 5:**
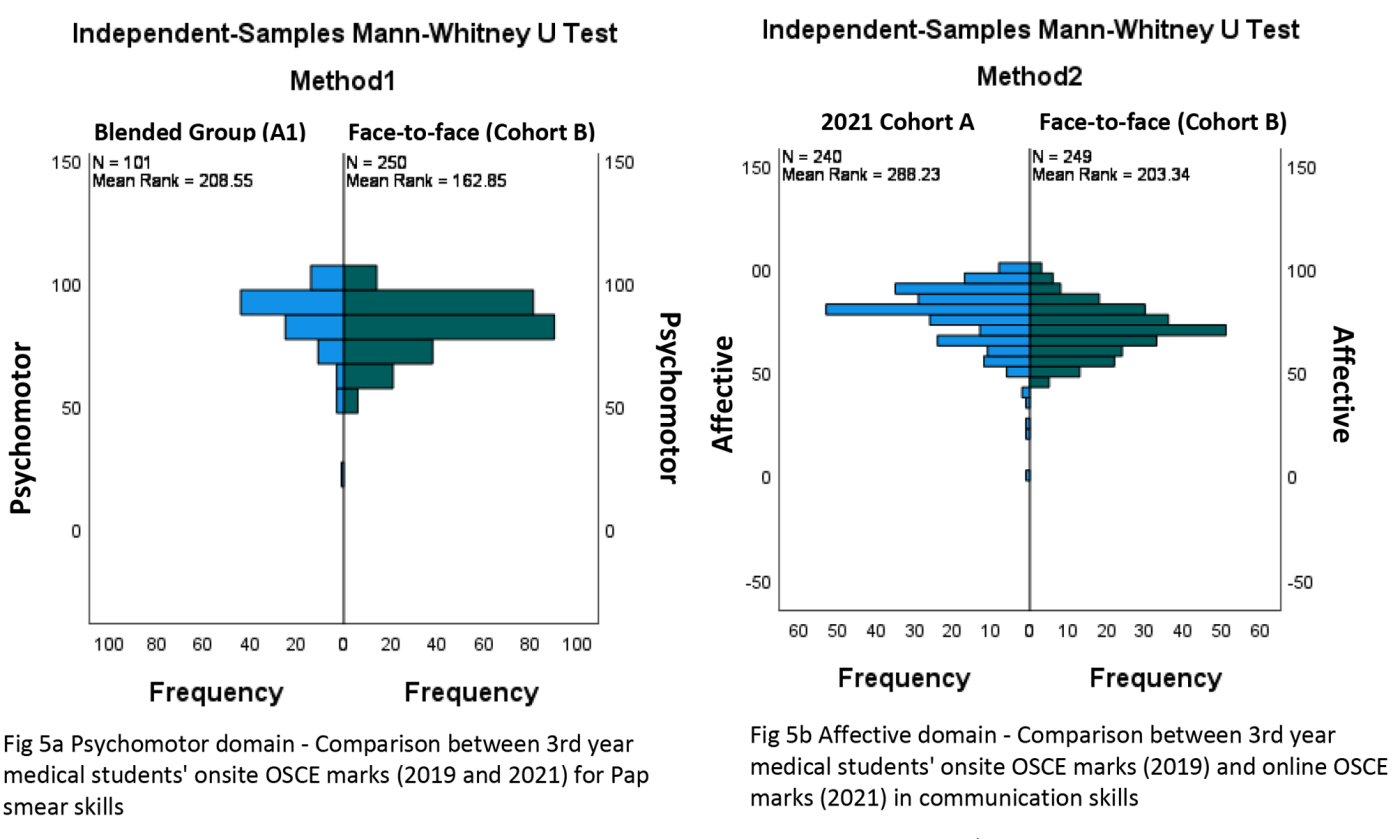
Methods 1 and 2 comparing Cohort A (2021) and Cohort B (2019) 3rd year OSCE scores in psychomotor and affective skills

The significance of this result was established by comparing the blended group of learners to their pre-pandemic, face-to-face counterparts, who performed the pap smear skill in 2019 in a summative OSCE. The blended group achieved a median of 90% compared to the face-to-face students, who achieved a median of 84% (Table 3). Figure 5 describes two methods used to analyse the learning and assessments conducted in both years. In method 1, Group A1 students performed significantly better in transferring skills from the online platform to the onsite platform than Cohort B students who were taught and assessed onsite (p < 0.05) (Fig. 5a). Method 2 is discussed below.

### Summative Online OSCE

The 2021 online summative OSCE assessed the affective and cognitive domains of clinical skills.

#### b) Affective Domain

Cohort A’s online history taking OSCE median score was 80%, 95%CI [78, 81], indicating near transfer of affective skills as the learning and assessment were on the same platform. Groups A1 and A2 had median scores of 82%, 95%CI [80, 85] and 78%, 95%CI [73, 79], respectively (p < 0.05). The blended group performed better than the e-learning group (p < 0.05), as illustrated in Table 3.

The median difference of the results for this domain was established by comparing Cohort A to Cohort B, who performed the same skill in 2019 (Fig. 5b). Method 2 describes learning and assessment being online for Cohort A and onsite for Cohort B. Cohort A’s median was 80% 95%CI[78, 81] and significantly better than Cohort B, whose median was 70% 95%CI[69.00, 72] (p < 0.05) (Table 3).

#### c) Cognitive Domain

Cognitive skills were demonstrated by applying clinical reasoning to patient scenarios based on examination and procedural skills. Cohort A’s median score for the online summative OSCE was 80%, 95%CI [78, 85]. Groups A1 and A2 achieved medians of 87%, 95% CI [84, 90] and 76%, 95%CI [71, 78], respectively, indicating near transfer as the learning and assessment were on the same platform. Group A1 performed better than Group A2 (p < 0.05) (Table 3).

**Table 3:** Results of Cohort A [2021 3rd Year Summative Online OSCE (Group A1 and A2), & Formative Onsite OSCE (Group A1)], and Cohort B [2019 Summative Onsite OSCE]

	COHORT A		COHORT B	
Domain	**Group A1** Blended Group Median (IQR)	**Group A2** E-learning Group Median (IQR)	Face-to-face group Median (IQR)	p-value
Affective	82% (15)	78% (20)	70% (17)	p < 0.05
Cognitive	87% (17)	76% (23)	-	p < 0.05
Psychomotor	90% (12)	-	84% (14)	p < 0.05

## Discussion

The mandatory shift to online medical education expanded the scope of computer-mediated instruction, forcing medical educators to re-examine existing training methods for practical skills traditionally reserved for the CSL and bedside [21]. This shift created the unprecedented opportunity to assess online learning, blended learning, and face-to-face approaches to clinical training allowing the merits of all three approaches to be vetted. We found that our 2021 students were competent in the affective, cognitive, and psychomotor domains of clinical skills, which required different degrees of transfer for learning to occur. Furthermore, the integrated online and blended platforms produced superior results to the traditional teaching approaches in some respects.

### Psychomotor domain

The flipped classroom (FC), a type of blended learning, focuses on asynchronous, independent, student-centered, self-paced learning of foundational concepts in preparation for in-class application with the teacher [13, 45, 46]. According to the systematic review by Chen et al. (2017), there are inconsistencies regarding its effectiveness, especially in undergraduate medical education [45]. Our study, however, focused on accomplishing the educational purpose by blending online and face-to-face elements using various methods, including online synchronicity, virtual and in-person group work, and active learning with tutor-driven and student-driven engagement [47]. Though blended learning has received positive responses as an effective active learning strategy for theoretical knowledge, it has had a minimal role in clinical skills [2, 33, 38, 48]. Students in our study competently demonstrated psychomotor skills they had never previously attempted. The remote online teaching programme provided knowledge through asynchronous online self-directed pre-learning material, synchronous online lectures, interactive practical zoom sessions, video demonstrations, and quizzes. Learning hands-on skills like the obstetric examination and pap smear procedure on an online platform required degrees of far transfer for students to perform the skills competently. Competent execution of these skills in the CSL was interesting since skills requiring far transfer are more difficult to perform [20, 22]. Further, similar to the findings by George et al. [6], our study highlights the positive impact of blended learning as Group A1 students’ performance was significantly better compared to the 2019 face-to-face students (Cohort B). The latter performed the same skill in a traditional summative onsite OSCE. Although OSCEs are considered less stressful than other examinations [49], the summative assessment may have impeded the 2019 students’ performance due to examination nervousness and anxiety. The 2021 students’ assessment was formative; however, the procedures assessed were technically more challenging for these students taught online due to the lack of opportunity and equipment required for self-directed practice. Perhaps the instructional teaching design, which was deliberately tailored to the online platform by employing novel home simulations, may have contributed to the higher performance in the blended group. Offiah et al. [50] and Anderson & Warren [26] found that simulation-based training is a successful online and onsite instructional technique that enhances learning. The psychomotor results achieved by Group A1 support Lala et al. [21], who described the blended learning teaching model as improving bedside training and essential clinical skills training. Aspects of online learning are possibly superior to traditional learning and bridge the gap between the textbook and the “hands-on” application of learned skills [21, 51].

Our study showed that using an online platform, with quality adaptations to teaching on par with traditional methods and learning process integration, could effectively train students for performance-based clinical skills requiring far transfer.

### Affective domain

The 2021 learners (Cohort A) demonstrated competence in the affective domain. Although the OSCE’s clinical case was changed, the elements in the initial teaching context were nearly identical to the exam setting, facilitating the evaluation of near transfer. This increased the likelihood that learners would perceive the two scenarios as comparable, resulting in improved transfer [19].

While online proctoring can be challenging [52], our study found that the directly assessed history-taking scenario was dynamic, requiring students to interact and actively demonstrate process skills and develop interpersonal relationships. This included showing empathy, emotions, and an existing knowledge base while gathering information from the simulated patient and clinically reasoning through the process.

Comparatively, Cohort A outperformed Cohort B, with the e-learning group (A2) performing better than the face-to-face group (Cohort B) in the history-taking skill. A possible reason is that virtual simulation-based training using the Zoom online platform enabled interactive small group teaching that facilitated the effective transfer of communication skills [6, 53]. Unlike onsite teaching, the continuous active learning strategies via Zoom’s online chats, polls, and breakout rooms ensured the engagement of all students during teaching sessions. The blended learners (A1) performed the best, despite receiving the same online instruction as Group A2. Shahrvini et al. [54] reported that students could perceive online learning as isolated due to a lack of connection to their colleagues and the institution, resulting in increased anxiety. The in-person interaction of the blended group (Group A1) with tutors at the formative OSCE may have reduced their anxiety, resulting in their higher scores. Our study thereby confirmed suggestions by Prober & Khan [16] that interactive and collaborative activities that reinforce the constructivist model could exceed the expectations of the learner using the online platform [55].

### Cognitive skills

In the traditional OSCE, the emphasis of the psychomotor domain was the technical demonstration of a physical examination or procedural skill. A clinical reasoning question was asked at the end of these performance-based skills and comprised approximately 15% of the overall examination score. For the online tm-OSCE, intellectual skills such as establishing a knowledge base, problem-solving, and critical thinking were examined as a viva voce in two five-minute scenarios and comprised 100% of the scores. Despite this novel component, students displayed adequate clinical knowledge retention when reasoning through procedural and examination-related cases. Students explained, defined, and rationalised the purpose of these skills, demonstrating near transfer of abilities since the assessment setting was similar to the learning environment [22].

Further analysis of the assessment scores revealed that the blended group, who practiced examination and procedural skills onsite, outperformed the e-learning group. This finding supports Turk et al. [56], who reported that combining online teaching and onsite performance may be preferable to online teaching alone.

Student characteristics, learning design, and onsite environmental conditions are also aspects to consider for the performance gap between the two groups [18]. Since the blended student group had volunteered for the onsite session, they may be more self-motivated. The formative OSCE was also preparation for the summative examination implying spaced learning [22]. The structure of the formative assessment allowed students to have one-on-one tutor interaction, where techniques were corrected and questions answered. Furthermore, the onsite practice allowed students to construct the applied skills on their existing knowledge, which is crucial in developing competence [18, 57, 58].

Despite the differences between the subgroups, Cohort A’s overall performance meant that most students could have a meaningful discussion with the examiner, demonstrating clinical reasoning and knowledge transfer [59]. Compared to the pre-pandemic onsite OSCE, the online viva voce examined a more significant proportion of the cognitive domain. Knowledge of clinical skills, emphasised in Step 4 of the adaptations as “Comprehension”, is essential for proficient performance [60]. According to Remmen et al. (2001), written tests are feasible alternatives to demonstration-based testing and can predict the student’s performance in the OSCE. Early exposure to understanding concepts coupled with CSL training and assessments may lead to better diagnostic reasoning, information retention, and student preparation for hospital rotations [16, 60]. Since online cognitive skills training went beyond the face-to-face scope, combining an e-learning platform with traditional teaching and assessment methods can potentially produce better outcomes [51].

Our study showed that medical students taught clinical skills on an online platform can effectively retain knowledge and transfer affective, cognitive, and psychomotor skills competently, bridging the theory-practice gap in three domains of clinical skills. Using a variety of blended teaching delivery approaches that extend beyond the scope of the FC and arranging immediate application opportunities, with support from clinical educators, could explain the higher-level transfer of assessed skills [6]. The improved transfer to “hands-on” practice petitions a revised blended-teaching strategy designed at the planning stage of the academic curriculum. The advantage of incorporating the tutor-driven blended adaptations and the student-driven FC is that it efficiently promotes a high-quality application of skills. Besides the academic advantages, online learning also allows students to build their skills and confidence before interacting with simulated or actual patients and other medical professionals [13]. Finally, in resource-constrained training contexts, the documented benefits of the online platform regarding time management, flexibility, and cost-effectiveness [38, 54, 56] could mean that more students can be included and trained.

## Limitations

The “Readiness Program” coincided with the fourth wave of the COVID-19 pandemic in South Africa, resulting in lower student participation. The non-randomisation of participants in this quasi-experimental study may have resulted in the more motivated and academically proficient students volunteering for the RP (Group A1). Additionally, as the online tm-OSCE was in a pilot phase, examiners were limited with training required to facilitate this exam. Further, with fewer examiners and larger class sizes, time constraints were a concern resulting in only three skills in each OCSE station being examined. However, measures were taken to ensure the assessment quality control, as discussed earlier. Although the findings demonstrated that students did transfer knowledge and skills from the online platform, a comprehensive summative onsite OSCE assessment of the entire class would better reflect the scale of far transfer. The ultimate test of competency would be to evaluate students at the bedside of patients.

## Conclusion and recommendations

The COVID-19 pandemic created an unprecedented opportunity to pilot an online approach to medical education and compare its impact on blended and face-to-face learning. The blended learning group performed significantly better in all clinical skill domains. Furthermore, our 2021 online learners bridged the theory-practice gap effectively and demonstrated higher-level transfer and knowledge retention than the 2019 face-to-face learners. Medical education needs to advocate a structural shift [61] to decentralise the classroom and provide an efficient and economical learning environment [48] that accommodates large cohorts [62]. Technology offers flexibility, small group work, and resource access that reinforces a constructivist model. Incorporating a digital curriculum that uses these varied instructional designs and assessments may support traditional teaching methods. Our study corroborates the findings of the blended learning literature and extends the current knowledge to clinical skills training. The contributing factors need to be critically analysed to implement a hybrid-teaching delivery model that formalises the gains made during this unique experience. A conceptual framework that optimises learning by amalgamating the best of the online and face-to-face platforms may evolve medical education while simultaneously minimising the impact of occurrences that can threaten the normal functioning of the physical institution.

## Data Availability

The datasets used or analysed during the current study are available from the corresponding author upon reasonable request.

## List of abbreviations

COVID-19: Coronavirus Disease 2019
CSL: Clinical skills laboratory
CCG: Calgary-Cambridge Guide
RP: Readiness Programme
FC: Flipped classroom
